# Supplementary material for: Hemoglobin point-of-care testing in rural Gambia: Comparing accuracy of HemoCue and Aptus with an automated hematology analyzer
Source: PLoS One. 2020 Oct 1;15(10):e0239931. doi: 10.1371/journal.pone.0239931 (PMC7529235; doi:10.1371/journal.pone.0239931)
Supplement: S1 Macro — Macro used in the study to calculate the Lin’s coefficient in SPSS. Available from: https://gjyp.nl/marta/Lin.sps. Date accessed: 23.04.2019. (DOCX) [file pone.0239931.s002.docx]

DEFINE LINCCC(!POS=!TOKENS(1)/!POS=!TOKENS(1)).

DATASET NAME OriginalData.

DATASET DECLARE Results WINDOW=HIDDEN.

MATRIX.

PRINT /TITLE="Lin's Concordance Coefficient".

GET pair

/VARIABLES=!1 !2

/NAMES=vname

/MISSING OMIT.

COMPUTE n=NROW(pair).

COMPUTE mean=CSUM(pair)/n.

COMPUTE variance=(CSSQ(pair)-n*mean&**2)/(n-1).

COMPUTE Minval=0.9*MMIN(pair).

COMPUTE Maxval=1.1*MMAX(pair).

PRINT {mean;variance}

/TITLE='X & Y Statistics'

/FORMAT='F8.3'

/RLABELS='Mean','Variance'

/CNAMES=vname.

PRINT n

/TITLE='Nr. of valid pairs'

/FORMAT='F8.0'.

COMPUTE covxy=((T(pair(:,1))*pair(:,2))-n*mean(1)*mean(2))/(n-1).

COMPUTE r=covxy/SQRT(variance(1)*variance(2)).

COMPUTE ncovxy=covxy*(n-1)/n.

COMPUTE nvarx=variance(1)*(n-1)/n.

COMPUTE nvary=variance(2)*(n-1)/n.

COMPUTE sdx=SQRT(nvarx).

COMPUTE sdy=SQRT(nvary).

COMPUTE lcc=2*ncovxy/(nvarx+nvary+(mean(1)-mean(2))**2).

COMPUTE cb=lcc/r.

COMPUTE zr=.5*LN((1+lcc)/(1-lcc)).

COMPUTE u=((n-1)/n)*(mean(1)-mean(2))/(SQRT(sdx*sdy)).

COMPUTE u2=u**2.

COMPUTE u4=u**4.

COMPUTE r2=r**2.

COMPUTE rc2=lcc**2.

COMPUTE rc3=lcc**3.

COMPUTE rc4=lcc**4.

COMPUTE term1=((1-r2)*rc2)/((1-rc2)*r2).

COMPUTE term2=(2*(rc3-rc4)*u2)/(r*((1-rc2)**2)).

COMPUTE term3=(rc4*u4)/(2*r2*((1-rc2)**2)).

COMPUTE sezr=SQRT((term1+term2-term3)/(n-2)).

COMPUTE lowz1=zr-1.645*sezr.

COMPUTE lowzr=zr-1.959964*sezr.

COMPUTE uppzr=zr+1.959964*sezr.

COMPUTE lowr1=((exp(2*lowz1))-1)/((exp(2*lowz1))+1).

COMPUTE lowr=((exp(2*lowzr))-1)/((exp(2*lowzr))+1).

COMPUTE uppr=((exp(2*uppzr))-1)/((exp(2*uppzr))+1).

PRINT {covxy,r}

/TITLE='Association statistics'

/FORMAT='F8.4'

/CLABELS="Cov.","R".

PRINT {lcc,cb,ABS(u)}

/TITLE="Lin's Concordance Coefficient & related statistics"

/CLABELS='Rc','Cb','Mn.Shift'

/FORMAT='F8.3'.

PRINT {lowr,uppr}

/FORMAT='F8.3'

/CLABELS='Lower' 'Upper'

/TITLE='95%CI for Rc'.

PRINT lowr1

/FORMAT='F8.3'

/TITLE='Lower one-sided 95% CL for Rc'.

PRINT {zr,sezr}

/FORMAT='F8.3'

/CLABEL='Z','SE(Z)'

/TITLE='Fisher transformation:'.

SAVE {Minval,Maxval} /OUTFILE=Results.

END MATRIX.

DATASET ACTIVATE Results.

PRESERVE.

SET LOCALE=ENGLISH.

STRING #var1 #var2 (A12).

DO REPEAT A=#var1 #var2 /B=col1 col2.

- COMPUTE A = STRING(B,E11.3).

END REPEAT.

!LET !minval=!UNQUOTE(#var1).

!LET !maxval=!UNQUOTE(#var2).

* Write Template to Temp *.

WRITE OUTFILE 'C:\Temp\LinTemplate.sgt'

/'<?xml version="1.0" encoding="UTF-8" standalone="no"?>'

/'<template SPSS-Version="1.4" date="2009-04-02" description="" selectPath="2 12 13 31 900 " '

/'xmlns="http://xml.spss.com/spss/visualization" '

/'xmlns:xsi="http://www.w3.org/2001/XMLSchema-instance"'

/'xsi:schemaLocation="http://xml.spss.com/spss/visualization '

/'http://xml.spss.com/spss/visualization/vizml-template-3.0.xsd">'

/' <setAxisStyle categorical="false" role="x">'

/' <style color="#000000" stroke-width="0.5pt" visible="true"/>'

/' </setAxisStyle>'

/' <setAxisStyle categorical="false" role="y">'

/' <style color="#000000" stroke-width="0.5pt" visible="true"/>'

/' </setAxisStyle>'

/' <addFrame count="1" type="visualization">'

/' <location bottom="500px" left="0px" right="500px" top="0px"/>'

/' <style color="#ffffff" color2="transparent" number="0" visible="true"/>'

/' <style font-family="SansSerif" font-size="8pt" number="1" pattern="0" stroke-linecap="butt" text-fit="true" visible="true"/>'

/' </addFrame>'

/' <setAxisInterval lowerMapping="exact" max="' !maxval '" min="' !minval '" role="x" upperMapping="exact"/>'

/' <setAxisInterval lowerMapping="exact" max="' !maxval '" min="' !minval '" role="y" upperMapping="exact"/>'

/' <addReferenceLine numberPoints="5" styleOnly="false" y="1 * x + 0" ycategorical="false">'

/' <style visible="true"/>'

/' </addReferenceLine>'

/' <addFrame count="1" styleOnly="true" type="graph">'

/' <style color="transparent" color2="transparent" visible="true"/>'

/' <style color="#ffffff" color2="#000000" number="1" visible="true"/>'

/' </addFrame>'

/' <setStyle subtype="simple" type="scatter">'

/' <style color="#000000" color2="#000000" size="4px" symbol="circle" visible="true"/>'

/' </setStyle>'

/'</template>'.

EXE.

DATASET ACTIVATE OriginalData.

DATASET CLOSE Results.

GRAPH

/SCATTERPLOT(BIVAR)=!1 WITH !2

/TEMPLATE='C:\Temp\LinTemplate.sgt'.

RESTORE.

!ENDDEFINE.

* Sample dataset *.

DATA LIST LIST /var1 var2 (2 F8.0).

begin data

END DATA.

LINCCC var1 var2.
