# Supplementary material for: Hemoglobin point-of-care testing in rural Gambia: Comparing accuracy of HemoCue and Aptus with an automated hematology analyzer
Source: PLoS One. 2020 Oct 1;15(10):e0239931. doi: 10.1371/journal.pone.0239931 (PMC7529235; doi:10.1371/journal.pone.0239931)
Supplement: S1 Table — Measurements in the Aptus were conducted in blood drops taken from a venous blood sample. Al HCT values given as percentage. The mean difference between the methods is 5.11 (95% CI: 4.76, 5.45) %, which was statistically significant (p<0.0001). (DOCX) [file pone.0239931.s003.docx]

|  | Aptus | Medonic |
| --- | --- | --- |
| Number of values | n=179 | n=179 |
|  |  |  |
| Minimum | 25 | 21.9 |
| 25% Percentile | 32 | 26.8 |
| Median | 34 | 28.8 |
| 75% Percentile | 36 | 30.5 |
| Maximum | 47 | 38.6 |
|  |  |  |
| Mean | 33.89 | 28.79 |
| Std. Deviation | 3.31 | 2.68 |
| Std. Error of Mean | 0.25 | 0.20 |
|  |  |  |
| Lower 95% CI | 33.41 | 28.39 |
| Upper 95% CI | 34.38 | 29.18 |
