# Supplementary figures and images for: Hemoglobin point-of-care testing in rural Gambia: Comparing accuracy of HemoCue and Aptus with an automated hematology analyzer
Source: PLoS One. 2020 Oct 1;15(10):e0239931. doi: 10.1371/journal.pone.0239931 (PMC7529235; doi:10.1371/journal.pone.0239931)

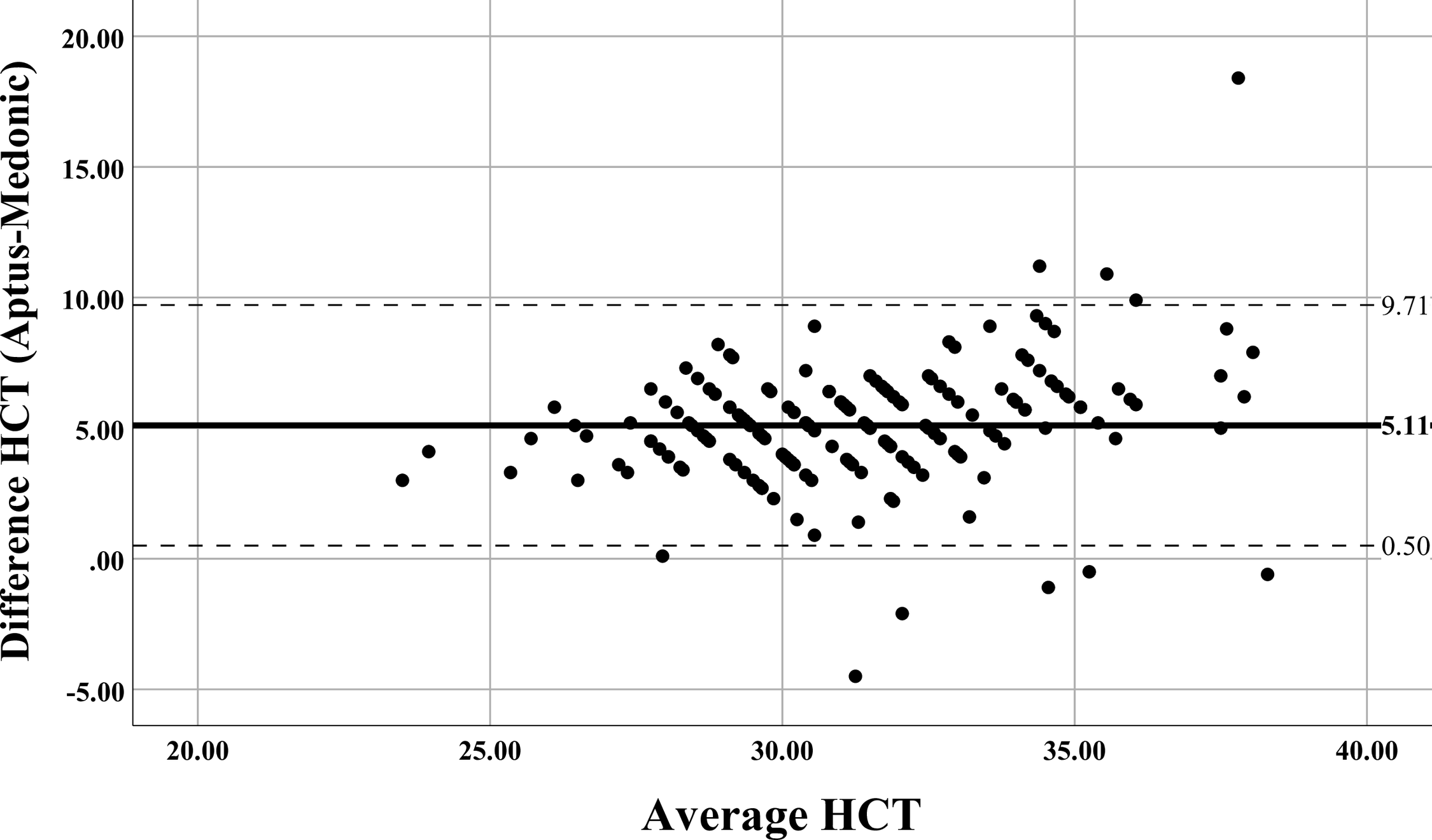

Supplement: S1 Fig — Data represents the difference (Aptus–Medonic®) versus the average HCT results. The solid lines represent the mean difference between the measurements (bias) is 5.1%, while the dotted lines indicate the 95% limits of agreement between methods of 0.5(lower) and 9.71% (upper) limit. (TIF) [file pone.0239931.s004.tif]

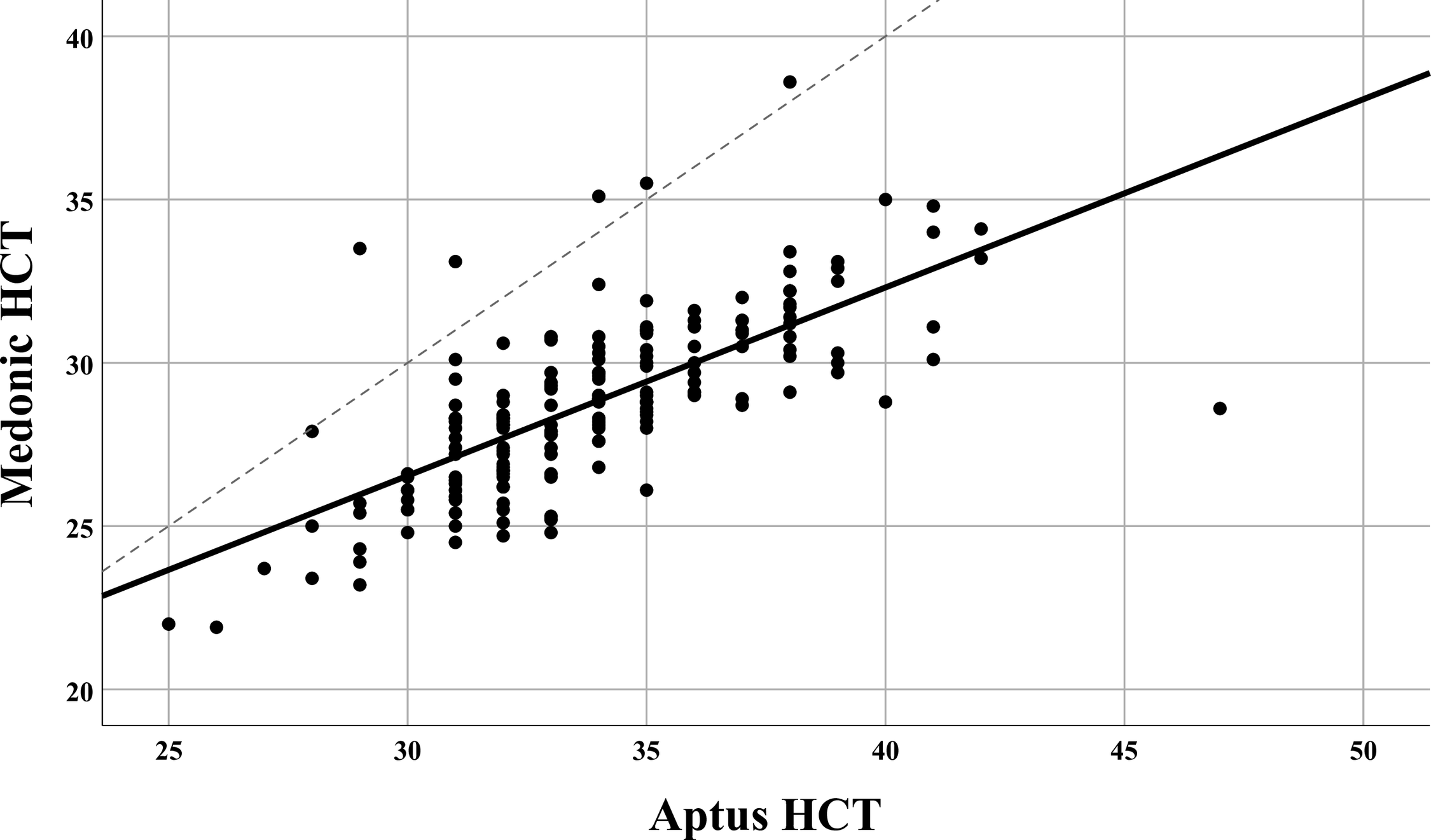

Supplement: S2 Fig — The dotted line represents the 45-degree line of perfect agreement through the origin, while the solid blue line is the line of best fit. Lin’s coefficient is rc = 0.285, and there is significant divergence from the 45-degree line and spread around the line of best fit (S2 Fig). It must be noted that the Aptus® does not provide decimals for HCT while the Medonic® does. This explains the columnar nature of the data in both plots, and negatively influences agreement due to an inherently lower accuracy of the Aptus®’s output. (TIF) [file pone.0239931.s005.tif]
