# Supplementary material for: Hemoglobin point-of-care testing in rural Gambia: Comparing accuracy of HemoCue and Aptus with an automated hematology analyzer
Source: PLoS One. 2020 Oct 1;15(10):e0239931. doi: 10.1371/journal.pone.0239931 (PMC7529235; doi:10.1371/journal.pone.0239931)
Supplement: Attachment — Submitted filename: Response to Reviewers.docx [file pone.0239931.s006.docx]

Dear editors,

We would like to thank the reviewers for their work and have addressed their concerns in the manner described below.

**Reviewer #1:**

**This study aimed to (1) compare Hb concentrations measured in venous blood by 2 PoCDs with an automated analyzer in young children with IDA in a rural setting (2) compare the Hb measurements provided by 2 PoCDs in capillary blood; (3) determine the usability of Aptus in these settings and 4) highlight potential improvements to the Aptus model used here before a wider global launch.

1) The method used for assessing the last two objectives are not well described in article under the "Materials and Method" section**

Clarification has been added in lines 98 – 102

**2) The abstract does not include the last 2 aims in it's objectives, nor the results but seems to jump to a conclusion on the use of one PoCD**

Descriptions have been added to the abstract (lines 8, 11, 20,21,23-24) **3) Statistical analysis: It is mentioned in the paper that "The Clinical Laboratory Improvement Amendments (CLIA) of 1988 postulate a ±7% deviance from the true value as acceptable for a device measuring hemoglobin." I think the limit of 7% is very large for hemoglobin measurements. I wish to suggest to the authors to use the total error allowable (TEa) based on** **Desirable Biological Variation Database specifications. For Hb, this is 4.19%.(**[**https://www.westgard.com/biodatabase1.htm#de**](https://www.westgard.com/biodatabase1.htm#de)**). This would be more fair method for comparing.**

The CLIA method has been replaced with the proposed TEa method (lines 292-295) **4) I wish to suggest the addition of ROC curves for the 2 PoCDs versus Lab measurements in addition to the agreement analysis**

A ROC analysis was added with absence of anemia (Hb ≥ 11g/dl) as binary outcome variable (lines 316 – 326). As explained in the text, severe and moderate anemia were not suitable for comparison due to the low prevalence.

**5) The manuscript should follow the Standards for Reporting of Diagnostic Accuracy studies - STARD statement (**[**http://www.stard-statement.org/**](http://www.stard-statement.org/)**) for properly reporting this type of study. A STARD checklist should ideally accompany this manuscript.**

Aspects of the text have been adapted to follow STARD guidelines and the checklist has been uploaded separately. We stress, however, that this was an exploratory comparative study and as such the intention was not to exhaustively determine the diagnostic accuracy of the PoCDs. Furthermore, the ancillary nature of this study necessitated adherence to IHAT-GUT study protocol, which was not planned as a comparative study between the devices and as such did not always represent the optimal study design for such a study.

**6) I think the Conclusion on the usability of Aptus at the end of this manuscript is not fully supported by the results of this study.**

In order to address this, the usage experience with the Aptus device has been moved from the Discussion to the Results section (lines 353-373), and the Discussion adapted to more clearly highlight the combination of statistical analysis and user experience that our conclusion is based on.

**7) This study included children. Please kindly specify whether consent was obtained from parents/legal guardians before study procedures**

Consent details have been specified (lines 132-143)

**Reviewer #2:**

**The authors of this work present an interesting work which evaluates the performance of 2 Point-of-care devices (PoCDs), Aptus® and HemoCue® against an automated haematology analyser in children in a country with limited resources, The Gambia. These 2 devices showed similar level of agreement with the laboratory analyser but Aptus® showed slightly lower bias and wider limits of agreement than the HemoCue®. Both PoCDs were shown to overestimate Hb concentration. Then, Aptus® could represent an alternative to HemoCue® as standard for field settings in low resource areas. The study design is clear and the results well presented. I agree with the conclusions and recommendations of this work. However, some clarifications will help to improve the quality of this work.

Introduction
1) The introduction is clear but would benefit from being more concise.**

Parts of the Introduction have been reformulated in a more concise way, see tracked changes.

**2) Table 1 (line 104): would it be possible to have an idea on the prices of these PoCDs, in order to be in agreement with the comment presented on line 369?**

Prices have been added to the last row of table 1 (line 104).

**Materials and methods
3) Why the choice of 35 months as the upper limit for the recruitment of children, and not 36 months, even 59 months as in the WHO report (The Global Prevalence of Anaemia in 2011)?**

Use of 35 months cut-off (inclusive, we could also write 6 to <36 months) is because this inclusion criteria was for children under the age of 3 years old which has been the group used in the iron supplementation trials conducted in sub-Saharan Africa showing a potential detrimental effect of iron supplementation in infection and diarrhea. We could have used the under 5s as defined by WHO (up to 59 months) but the trials with iron have been focusing on under 35months. Furthermore, after 3 years the microbiome changes too close to an adult-like microbiome and impact of iron on microbiome was an important endpoint of IHAT-GUT and the hypothesis behind IHAT. You can reference:

<https://pubmed.ncbi.nlm.nih.gov/16413877/>

and

<https://www.sciencedirect.com/science/article/pii/S0966842X19302148>

**4) Children with severe anaemia were excluded from this study (line 124). For children between 6 and 59 months of age, WHO defines severe anaemia as <7.0 g / dL. Can you explain why in the basic characteristics of the participants in your study, the minimum haemoglobin level is 6.9 g / dL? (Table 2). Same as in Table 3 where the minimum Hb level for Medonic is 6.7 g / dL?**

Children had Hb ≥7 g/dL measured by the Medonic at the time of screening and enrolment in the study. The data presented in this manuscript was collected from study day 57 onwards, at which point some children had shown a drop of Hb to below the severe anemia cut-off. These children were subsequently discontinued of the study supplementation, as per IHAT-GUT study protocol, and given standard of care iron drops according to national guidelines in The Gambia. As this clinical consequence does not interfere with the methodological comparison of the measuring devices, these measurements were not excluded in the present study.

For clarification, this explanation was added to the Methods section (lines 170-179, 217-222)

**Results
5) Do you have climate data (particularly the temperature) at the time the blood samples were taken and the analysis of these samples was performed?**

Temperature and humidity averages added (line 110,111).

**Discussion
6) The discussion is clear but would benefit from being more concise.**

Parts of the Discussion have been reformulated in a more concise way, see tracked changes. Furthermore, the description of the Aptus in-field usage has been moved to the Results section (lines 353-373), thereby significantly shortening the Discussion.

**Academic Reviewer
1. PLOS ONE's style requirements, including those for file naming.**

File names have been amended, Panels in figures 2-4 combined into planes, line numbers added to the title page and minor formatting issues fixed in the main text.

**2. Competing interests and funding statements**

Competing interests and funding statements have been amended and clarified as per the feedback received.

**3. Figures**

All figure files have been checked with Preflight Analysis and Conversion Engine (PACE) digital diagnostic tool.

We believe the manuscript is now suitable for publication in your journal.

Kind regards,

Stefan Nass

On behalf of all the authors
