# Supplementary material for: Hemoglobin point-of-care testing in rural Gambia: Comparing accuracy of HemoCue and Aptus with an automated hematology analyzer
Source: PLoS One. 2020 Oct 1;15(10):e0239931. doi: 10.1371/journal.pone.0239931 (PMC7529235; doi:10.1371/journal.pone.0239931)
Supplement: Attachment — Submitted filename: Response to Reviewers.docx [file pone.0239931.s007.docx]

Dear editors,

We would like to thank the reviewers for their work and have addressed their concerns in the manner described below.

**Reviewer #1:**

**1) A considerable amount of grammatical errors noted with additional "the", "a", sentences which are too long making their comprehension difficult**

The manuscript has been reviewed again by three native speakers. For the revisions, see tracked changes.

**4. Have the authors made all data underlying the findings in their manuscript fully available? Answer: No**

The reviewer has not further specified which data they are referring to. All data pertaining to this analysis is presented in the manuscript. The demographic and endpoint data for the IHAT-GUT trial will be published separately and the full database for the IHAT-GUT trial will be made available upon publication of the primary and secondary endpoints. We trust that this is sufficient with regards to the data policy.

We believe the manuscript is now suitable for publication in your journal.

Kind regards,

Stefan Nass

On behalf of all the authors
